# Long-Term Follow-Up of a Patient with Ankylosis of a Primary Incisor Caused by Trauma: A Case Report

**DOI:** 10.3390/reports8010027

**Published:** 2025-02-26

**Authors:** Tatsuya Akitomo, Shuma Hamaguchi, Chieko Mitsuhata, Ryota Nomura

**Affiliations:** Department of Pediatric Dentistry, Graduate School of Biomedical and Health Sciences, Hiroshima University, Hiroshima 734-8553, Japan; syuumai@hiroshima-u.ac.jp (S.H.); chiekom@hiroshima-u.ac.jp (C.M.); rnomura@hiroshima-u.ac.jp (R.N.)

**Keywords:** ankylosis, replantation, primary incisor, trauma

## Abstract

**Introduction and Clinical Significance:** Tooth ankylosis is a serious complication that can occur because of the replantation of an avulsed tooth. However, few reports have investigated the follow-up of replanted or ankylosed primary incisors because the replantation of primary teeth is not recommended in the guidelines of the International Association of Dental Traumatology. **Case Presentation:** A boy aged 4 years and 8 months was referred to hospital for further evaluation of the maxillary right primary central incisor. It had been avulsed and replanted 2 years earlier. The tooth was positioned higher than the central incisor on the left side, and a metallic percussion sound was noted, leading to a diagnosis of tooth ankylosis. Long-term follow-up revealed the progression of ankylosis, and the tooth was finally extracted. At the age of 7 years and 6 months, permanent tooth eruption was detected, and no pathological finding was observed. **Conclusions:** In this case, root resorption of the replanted primary incisor was observed with age, but tooth ankylosis progressed, and natural exfoliation was difficult. The authors extracted the primary incisor appropriately, which aided the eruption of a successor permanent tooth. This report suggests the importance of patients visiting the dentist regularly after trauma to primary teeth and appropriate treatment by dentists to erupt the permanent teeth.

## 1. Introduction and Clinical Significance

Tooth eruption can be disturbed as a result of malpositioning of the tooth bud, primary failure of eruption, medical syndromes, or dental abnormalities [1,2,3]. Tooth ankylosis can also disturb tooth eruption and may cause serious complications in growing individuals [4,5]. Ankylosis is an eruptive abnormality characterized by the fusion of the dentin or cementum of the root with the surrounding bone, with obliteration of the periodontal ligament, which is progressively replaced by bone tissue [6]. Although the etiology is still poorly understood, trauma is thought to be an important factor [7,8,9,10].

Dental avulsion, the complete displacement of a tooth from its socket, is a form of severe dental trauma with a prevalence of 0.5% to 16% of all dental injuries [11,12,13,14]. Although the replantation of teeth is an effective therapeutic approach for tooth avulsion, some cases result in tooth ankylosis [4,15,16,17,18]. There are some reports of tooth ankylosis occurring in permanent incisors as a result of trauma [19,20]. The guidelines of the International Association of Dental Traumatology recommend that avulsed primary teeth should not be replanted because of the burden for the patient or the impact on the permanent teeth [21]. However, children with missing anterior teeth are at risk of developing abnormal tongue habits, and the absence of anterior teeth may lead to mental disorders; therefore, replantation of these teeth has been recommended by some authors [22,23,24,25]. Although avulsed primary teeth can be replanted, the total number of cases is small and there have been no reports of subsequent tooth ankylosis.

The authors encountered a patient with a replanted primary incisor who subsequently developed tooth ankylosis. This report describes the long-term follow-up of the patient up until eruption of the permanent tooth.

## 2. Case Presentation

A Japanese boy aged 4 years and 8 months was referred to hospital with a chief complaint of tooth abnormalities. The maxillary right primary central incisor was avulsed following trauma at the age of 2 years and 6 months. He visited an urgent care clinic and the tooth was replanted. He continued having regular dental checkups with the family dentist; however, when ankylosis of the maxillary right central incisor was observed, the patient was referred to hospital. Although heart disease was noted, the patient’s medical doctor advised that antibiotic prophylaxis was not needed before invasive dental treatment. There was no other medical or family history.

**Figure 1:**
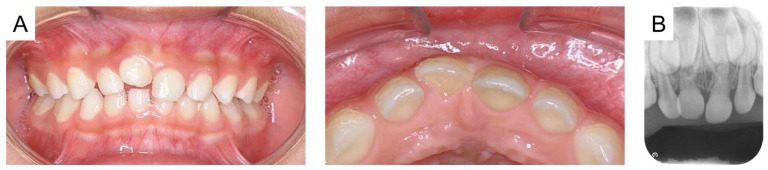
Initial examination at the age of 4 years and 8 months: (**A**) intraoral photographs and (**B**) periapical radiograph.

Intraoral photographs and radiographs are shown in Figure 1. The maxillary right primary central incisor was positioned higher than the central incisor on the left side, and a metallic percussion sound was noted. A periapical radiograph revealed external root resorption of the maxillary right primary central incisor, and the periodontal ligament appeared unclear. Additionally, the maxillary left primary central incisor exhibited external root resorption and obstruction of the pulp chamber. However, there was no difference in positioning between the unerupted maxillary permanent central incisors.

**Figure 2:**
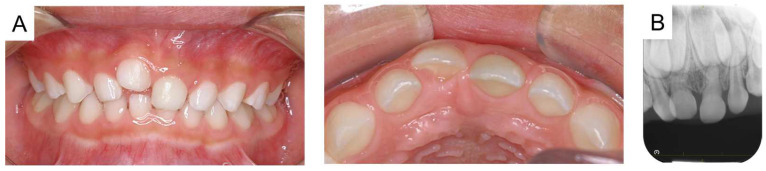
Images at the age of 5 years and 2 months: (**A**) intraoral photographs and (**B**) periapical radiograph.

**Figure 3:**
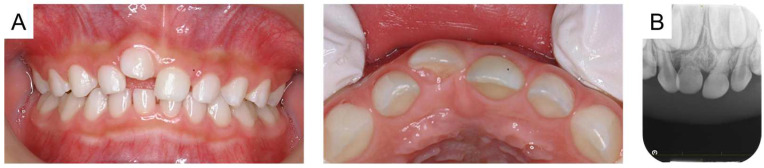
Images at the age of 5 years and 8 months: (**A**) intraoral photographs and (**B**) periapical radiograph.

**Figure 4:**
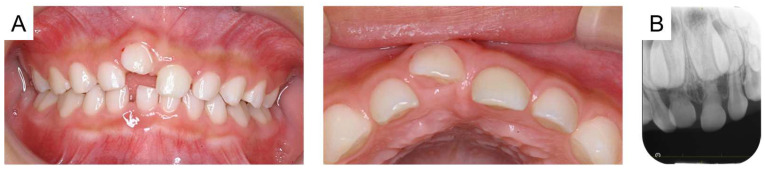
Images at the age of 6 years and 2 months: (**A**) intraoral photographs and (**B**) periapical radiograph.

The maxillary right primary central incisor was diagnosed as an ankylosed tooth caused by trauma, and the authors continued to monitor the tooth because there were no subjective symptoms or signs of inflammation. Time-lapse photographs of the oral cavity are shown in Figure 2, Figure 3 and Figure 4. The maxillary right primary incisor continued to move labially and rootward over time; however, there were no other notable events during the next 2 years.

**Figure 5:**
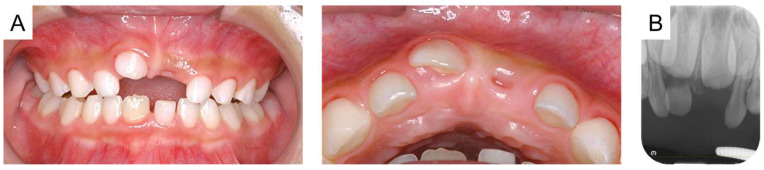
Images at the age of 6 years and 8 months: (**A**) intraoral photographs and (**B**) periapical radiograph.

**Figure 6:**
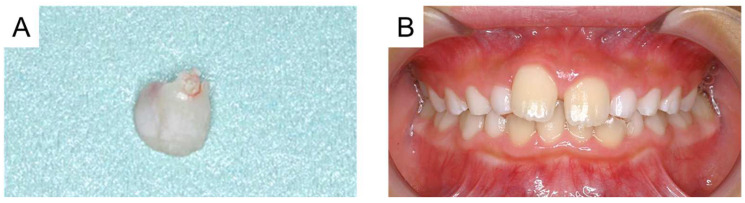
(**A**) Photograph of the maxillary right primary incisor at the age of 6 years and 11 months and (**B**) intraoral photograph at the age of 8 years and 2 months.

At the age of 6 years and 8 months, the maxillary left central incisor was shed, and intraoral examination showed slight mobility of the maxillary right primary central incisor. A periapical radiograph revealed physiological root resorption of the maxillary right primary central incisor (Figure 5). The maxillary right primary central incisor remained in place for the next 3 months and was then extracted (Figure 6A). The maxillary bilateral central permanent incisor erupted at 7 years and 6 months, and no abnormal findings such as enamel hypoplasia were observed at the age of 8 years and 2 months (Figure 6B).

## 3. Discussion

Dental professionals encounter a variety of patients, including those with dental caries and periodontal disease, as well as those who require surgical treatment for tumors or dental abnormalities, and those who require oral care while hospitalized [26,27,28,29,30,31]. Dental trauma is also common, and the reported prevalence is 24.2% in primary teeth [32]. There are two peaks of incidence in boys at 1–3 years and 10–12 years and one peak of incidence in girls at 1–3 years of age [11,33,34]. Dental trauma can be divided into fracture and luxation injuries and further classified according to severity [34]. Mendoza-Mendoza et al. (2015) reported that the most common injury in primary teeth is subluxation, followed by intrusion and avulsion [35]. Intrusion and avulsion often result in dental anomalies in successor permanent teeth; therefore, they are the most controversial with regard to definitive treatment, prognosis, and sequelae [36,37].

In the present case, the patient had a traumatic tooth injury, and the tooth was replanted at the age of 2 years and 6 months. When the patient was referred to hospital approximately 2 years later, the maxillary right primary central incisor was positioned higher than the central incisor on the left side. Replantation often results in poor outcomes with two major complications: tooth ankylosis and root resorption [18]. Ankylosis occurs when the periodontal ligament is damaged; the root surface and alveolar bone are fused, reducing the periodontal ligament tissue between them [4,18]. Additionally, the first sign of ankylosis is a high or metallic sound on percussion, followed by decreased mobility and replacement resorption. In the present case, tooth ankylosis was diagnosed following radiographic examination and the presence of a high percussion sound [4].

Immediate extraction is not routinely recommended for two reasons when a diagnosis of irreversible ankylosis is confirmed [38]. First, early extraction of an ankylosed tooth might be complicated by damage to the alveolar bone. Additionally, the tooth is frequently retained for several more years to provide the patient with an esthetic and functional solution [38,39]. Mohadeb et al. (2016) reported that the rate of replacement root resorption is more rapid in a growing patient, and the affected tooth crown will eventually fall off as it is deprived of root support [40]. Andersson et al. (1999) also reported that in patients younger than 10 years of age at injury, the increase in infraposition is fast and more severe than if the trauma had occurred later and concluded that this is because of the vertical growth of the maxilla during the eruption of the permanent incisors [41]. These reports concern permanent teeth, but pediatric patients with primary teeth are still growing, and there is a risk that ankylosis of the primary tooth may be severe. In the present case, as the patient had no other symptoms, the ankylosed tooth was monitored, resulting in progression of the severity of ankylosis. A relationship is established between the infraposition of the replanted ankylosed teeth and the age of the patient at the time of the injury [38,42,43]. Interestingly, the intraoral photographs show that the affected tooth moved labially and rootward during the next 2 years. As the maxillary dental arch grows in the incisor area, bone resorption occurs on the outer labial surface and bone formation occurs on the inner lingual surface [44]. The movement of the affected tooth over time in the present case correlated with the movement of the alveolar bone associated with maxillary remodeling, explaining the direction of tooth movement of ankylosed teeth during growth.

The ankylosed tooth should be monitored until it interferes with eruption of the succedaneous tooth or causes tipping of the adjacent teeth or supraeruption of the opposing teeth [45]. If tipping occurs, and space is lost, the tooth should be extracted and the space should be maintained until the permanent successor has erupted [45,46]. The authors continued to monitor the ankylosed tooth and no obvious eruption disturbance of the succendaneous tooth or tipping of the adjacent teeth were detected. A tooth that is definitely ankylosed may surprisingly undergo root resorption at a given time and be normally shed [47]. On the other hand, Tieu et al. (2013) reported that ankylosed primary molars should be monitored closely, and they should be removed if they do not exfoliate spontaneously [48]. In the present case, although radiographic examination at the age of 6 years and 8 months showed root resorption, the tooth was left in place and required extraction to enable eruption of the permanent tooth several months later. This suggests that it is difficult for an ankylosed primary tooth to exfoliate even in the presence of physiological root resorption and highlights the importance of monitoring the ankylosed primary tooth.

Some systemic diseases need to be considered when undertaking dental procedures. When performing surgical procedures on patients with hemophilia, coagulation factor replacement may be necessary to prevent excessive bleeding [49]. Additionally, patients with heart disease require antibiotic prophylaxis to prevent infective endocarditis [50]. In the present case, although the patient was diagnosed with heart disease, the medical doctor advised us that the patient did not require antibiotic prophylaxis. Dental professionals need to confirm the systemic condition of patients with tooth ankylosis because extraction may be required at regular dental checkups.

Dental trauma to a deciduous tooth can damage the bud of the permanent tooth, and enamel discoloration and/or hypoplasia are the most common sequelae [51]. Del Negro et al. (2022) concluded that the risk of sequelae in the permanent teeth after avulsion of the primary tooth is higher when the trauma occurs in young children (<2 years) [52]. In the present case, the dental trauma occurred at the age of 2 years and 6 months, and there were no abnormal findings over 6 years to indicate that the maxillary right permanent central incisor erupted slower compared to the left side. In addition, although ankylosis of the maxillary right primary central incisor occurred, it could be preserved until the tooth exfoliation period with monitoring of the tooth. Regular dental checkups are important for the early detection of disease, and continuous dental support leads to better general oral health, which improves patients’ quality of life [53,54]. The patient continues to have regular dental checkups at this clinic.

The most important thing for the replantation of primary teeth is to explain the condition to the patient and their guardians and to continue regular dental visits. In clinical practice, an accurate assessment of children’s decision-making capacity is needed to avoid two pitfalls: imposing complex medical decisions on children who are unable to make them and inadvertently excluding capable children who want to take part in decision-making [55,56]. Hein et al. (2015) reported that, generally, children of 11.2 years and above were competent, while children of 9.6 years and younger were not; therefore, in cases of trauma to primary teeth, the treatment decision is left to the guardians [56]. Although a permanent tooth germ traumatized at the pre-eruption stage frequently suffers various consequences, some can only be diagnosed radiologically. Therefore, it is important that pulpal and periodontal healing is monitored through clinical examination, mobility tests, and standardized radiological controls during the follow-up period [57,58]. If there is an abnormality, the replanted tooth may be extracted; in which case, a denture will be required to maintain the space. Additionally, some cases require surgical/orthodontic treatment when a permanent incisor is impacted [59,60]. Replantation of primary teeth has significant benefits regarding the aesthetic, social, and emotional function of keeping the teeth in the child’s mouth. On the other hand, tooth ankylosis or root resorption can occur, which are the disadvantages of the replantation of primary teeth. Additionally, when patients stop attending regular dental checkups, this can lead to delayed detection or treatment, as some issues can only be detected through radiographic examination. This may affect the eruption and preservation of successor permanent teeth, which are risks that must be considered. Dental professionals should provide this information to the patient and their guardians, and continuing long-term oral management in collaboration with them is essential.

This report has a limitation. The patient’s tooth was replanted not at a family dentist but in an urgent care clinic; therefore, detailed information was lacking about the dental trauma and the condition of the avulsed primary incisor. Coste et al. (2020) reported that older patient age at the time of trauma, the stage of root development, and the storage of avulsed teeth in milk during the extra-alveolar period were important prognostic factors for tooth survival after replantation [61]. Trope (1996) reported that the accident site, the amount of time the tooth is out of the socket, the storage medium, and the endodontic protocol all influence the prognosis [62]. Future investigations into the long-term prognosis of avulsed primary teeth may lead to the identification of prognostic factors for the replantation of primary teeth.

## 4. Conclusions

In this case, root resorption of the replanted primary incisor was observed with age, but tooth ankylosis progressed, and natural exfoliation was difficult. The authors extracted the primary incisor appropriately, which aided the eruption of a successor permanent tooth. This report suggests the importance of patients visiting the dentist regularly after trauma to primary teeth and appropriate treatment by dentists to erupt the permanent teeth.

## Data Availability

The original contributions presented in this study are included in the article. Further inquiries can be directed to the corresponding author.

## References

[1] Ducommun F., Bornstein M.M., Bosshardt D., Katsaros C., Dula K. (2018). Diagnosis of tooth ankylosis using panoramic views, cone beam computed tomography, and histological data: A retrospective observational case series study. Eur. J. Orthod..

[2] Akitomo T., Asao Y., Iwamoto Y., Kusaka S., Usuda M., Kametani M., Ando T., Sakamoto S., Mitsuhata C., Kajiya M. (2023). A Third Supernumerary Tooth Occurring in the Same Region: A Case Report. Dent. J..

[3] Akitomo T., Kusaka S., Usuda M., Kametani M., Kaneki A., Nishimura T., Ogawa M., Mitsuhata C., Nomura R. (2023). Fusion of a Tooth with a Supernumerary Tooth: A Case Report and Literature Review of 35 Cases. Children.

[4] Malmgren B. (2013). Ridge preservation/decoronation. Pediatr. Dent..

[5] Shi K.K., Kim J.Y., Choi T.H., Lee K.J. (2014). Timely relocation of subapically impacted maxillary canines and replacement of an ankylosed mandibular molar are the keys to eruption disturbances in a prepubertal patient. Am. J. Orthod. Dentofac. Orthop..

[6] Eşian D., Bica C.I., Stoica O.E., Dako T., Vlasa A., Bud E.S., Salcudean D., Beresescu L. (2022). Prevalence and Manifestations of Dental Ankylosis in Primary Molars Using Panoramic X-rays: A Cross-Sectional Study. Children.

[7] Rubin P.L., Weisman E.J., Bisk F. (1984). Experimental tooth ankylosis in the monkey. Angle Orthod..

[8] Donaldson M., Kinirons M.J. (2001). Factors affecting the time of onset of resorption in avulsed and replanted incisor teeth in children. Dent. Traumatol..

[9] Fuss Z., Tsesis I., Lin S. (2003). Root resorption—Diagnosis, classification and treatment choices based on stimulation factors. Dent. Traumatol..

[10] Zaleckiene V., Peciuliene V., Brukiene V., Drukteinis S. (2014). Traumatic dental injuries: Etiology, prevalence and possible outcomes. Stomatologija.

[11] Andreasen J.O., Andreasen F.M., Andersson L. (2019). Textbook and Color Atlas of Traumatic Injuries to The Teeth.

[12] Day P.F., Duggal M., Nazzal H. (2019). Interventions for treating traumatised permanent front teeth: Avulsed (knocked out) and replanted. Cochrane Database Syst. Rev..

[13] Fouad A.F., Abbott P.V., Tsilingaridis G., Cohenca N., Lauridsen E., Bourguignon C., O’Connell A., Flores M.T., Day P.F., Hicks L. (2020). International Association of Dental Traumatology guidelines for the management of traumatic dental injuries: 2. Avulsion of permanent teeth. Dent. Traumatol..

[14] Zerman N. (2024). Replantation After Dental Avulsion: A Scoping Review and Proposal of a Flow Chart. Eur. J. Paediatr. Dent..

[15] Andersson L., Lindskog S., Blomlöf L., Hedström K.G., Hammarström L. (1985). Effect of masticatory stimulation on dentoalveolar ankylosis after experimental tooth replantation. Endod. Dent. Traumatol..

[16] Malmgren B., Tsilingaridis G., Malmgren O. (2015). Long-term follow up of 103 ankylosed permanent incisors surgically treated with decoronation–a retrospective cohort study. Dent. Traumatol..

[17] Maslamani M., Almusawi A., Joseph B., Gabato S., Andersson L. (2016). An experimental model for studies on delayed tooth replantation and ankylosis in rabbits. Dent. Traumatol..

[18] Ideno H., Komatsu K., Nakashima K., Nifuji A. (2022). Tooth transplantation and replantation: Biological insights towards therapeutic improvements. Genesis.

[19] Kotsanos I.N., Tzika E., Economides N., Kotsanos N. (2023). Intentional replantation and management of avulsion related ankylosis and external cervical resorption. A 10-year follow up case report. Dent. Traumatol..

[20] Li K., Sun P., Sun J., Wang T. (2023). Combined orthodontic and prosthodontic treatment in an adolescent patient with traumatically ankylosed incisors: A case report. Dent. Traumatol..

[21] Day P.F., Flores M.T., O’Connell A.C., Abbott P.V., Tsilingaridis G., Fouad A.F., Cohenca N., Lauridsen E., Bourguignon C., Hicks L. (2020). International Association of Dental Traumatology guidelines for the management of traumatic dental injuries: 3. Injuries in the primary dentition. Dent. Traumatol..

[22] Filippi A., Pohl Y., Kirschner H. (1997). Replantation of avulsed primary anterior teeth: Treatment and limitations. J. Dent. Child..

[23] Weiger R., Heuchert T. (1999). Management of an avulsed primary incisor. Endod. Dent. Traumatol..

[24] de Carvalho Rocha M.J., Cardoso M. (2008). Reimplantation of primary tooth—Case report. Dent. Traumatol..

[25] Akitomo T., Kusaka S., Iwamoto Y., Usuda M., Kametani M., Asao Y., Nakano M., Tachikake M., Mitsuhata C., Nomura R. (2023). Five-Year Follow-Up of a Child with Non-Syndromic Oligodontia from before the Primary Dentition Stage: A Case Report. Children.

[26] Frencken J.E., Sharma P., Stenhouse L., Green D., Laverty D., Dietrich T. (2017). Global epidemiology of dental caries and severe periodontitis—A comprehensive review. J. Clin. Periodontol..

[27] Sun H.Y., Jiang H., Du M.Q., Wang X., Feng X.P., Hu Y., Lin H.C., Wang B., Si Y., Wang C.X. (2018). The Prevalence and Associated Factors of Periodontal Disease among 35 to 44-year-old Chinese Adults in the 4th National Oral Health Survey. Chin. J. Dent. Res..

[28] Rayamajhi S., Shrestha S., Shakya S., Bhandari S., Twayana A.R., Shahi K. (2022). Unicystic Ameloblastoma of Mandible: A Case Report. JNMA J. Nepal. Med. Assoc..

[29] Usuda M., Kametani M., Hamada M., Suehiro Y., Matayoshi S., Okawa R., Naka S., Matsumoto-Nakano M., Akitomo T., Mitsuhata C. (2023). Inhibitory Effect of Adsorption of *Streptococcus mutans* onto Scallop-Derived Hydroxyapatite. Int. J. Mol. Sci..

[30] Henninger E., Friedli L., Makrygiannakis M.A., Zymperdikas V.F., Papadopoulos M.A., Kanavakis G., Gkantidis N. (2023). Supernumerary Tooth Patterns in Non-Syndromic White European Subjects. Dent. J..

[31] Akitomo T., Tsuge Y., Mitsuhata C., Nomura R. (2024). A Narrative Review of the Association between Dental Abnormalities and Chemotherapy. J. Clin. Med..

[32] Patnana A.K., Chugh A., Chugh V.K., Kumar P., Vanga N.R.V., Singh S. (2021). The prevalence of traumatic dental injuries in primary teeth: A systematic review and meta-analysis. Dent. Traumatol..

[33] Petti S., Glendor U., Andersson L. (2018). World traumatic dental injury prevalence and incidence, a meta-analysis—One billion living people have had traumatic dental injuries. Dent. Traumatol..

[34] Tewari N., Bansal K., Mathur V.P. (2019). Dental Trauma in Children: A Quick Overview on Management. Indian. J. Pediatr..

[35] Mendoza-Mendoza A., Iglesias-Linares A., Yañez-Vico R.M., Abalos-Labruzzi C. (2015). Prevalence and complications of trauma to the primary dentition in a subpopulation of Spanish children in southern Europe. Dent. Traumatol..

[36] Wilson C.F. (1995). Management of trauma to primary and developing teeth. Dent. Clin. North. Am..

[37] Bardellini E., Amadori F., Pasini S., Majorana A. (2017). Dental Anomalies in Permanent Teeth after Trauma in Primary Dentition. J. Clin. Pediatr. Dent..

[38] Sapir S., Shapira J. (2008). Decoronation for the management of an ankylosed young permanent tooth. Dent. Traumatol..

[39] Malmgren O., Malmgren B., Goldson L., Andreasen J.O., Andreasen F.M. (1994). Orthodontic management of the traumatized dentition. Textbook and Color Atlas of Traumatic Injuries to the Teeth.

[40] Mohadeb J.V., Somar M., He H. (2016). Effectiveness of decoronation technique in the treatment of ankylosis: A systematic review. Dent. Traumatol..

[41] Andersson L., Malmgren B. (1999). The problem of dentoalveolar ankylosis and subsequent replacement resorption in the growing patient. Aust. Endod. J..

[42] Malmgren B. (2000). Decoronation: How, why, and when?. J. Calif. Assoc..

[43] Malmgren O., Malmgren B. (2002). Rate of infraposition of reimplanted ankylosed incisors related to age and growth in children and adolescents. Dent. Traumatol..

[44] Shirakawa T., Fukumoto S., Iwamoto T., Morikawa K. (2023). Pediatric Dentistry.

[45] Noble J., Karaiskos N., Wiltshire W.A. (2007). Diagnosis and management of the infraerupted primary molar. Br. Dent. J..

[46] Proffit W.R. (2000). Contemporary Orthodontics.

[47] Parisay I., Kebriaei F., Varkesh B., Soruri M., Ghafourifard R. (2013). Management of a severely submerged primary molar: A case report. Case Rep. Dent..

[48] Tieu L.D., Walker S.L., Major M.P., Flores-Mir C. (2013). Management of ankylosed primary molars with premolar successors: A systematic review. J. Am. Dent. Assoc..

[49] Kametani M., Akitomo T., Usuda M., Kusaka S., Asao Y., Nakano M., Iwamoto Y., Tachikake M., Ogawa M., Kaneki A. (2024). Evaluation of Periodontal Status and Oral Health Habits with Continual Dental Support for Young Patients with Hemophilia. Appl. Sci..

[50] Thornhill M.H., Gibson T.B., Durkin M.J., Dayer M.J., Lockhart P.B., O’Gara P.T., Baddour L.M. (2020). Prescribing of antibiotic prophylaxis to prevent infective endocarditis. J. Am. Dent. Assoc..

[51] Caeiro-Villasenín L., Serna-Muñoz C., Pérez-Silva A., Vicente-Hernández A., Poza-Pascual A., Ortiz-Ruiz A.J. (2022). Developmental Dental Defects in Permanent Teeth Resulting from Trauma in Primary Dentition: A Systematic Review. Int. J. Environ. Res. Public. Health.

[52] Del Negro B., Lauridsen E., Mendes F.M., Andreasen J.O., Wanderley M.T., Hermann N.V. (2022). Impact of avulsion of the primary incisors on the occurrence of sequelae in the permanent teeth: A retrospective cohort study. Community Dent. Oral. Epidemiol..

[53] Usuda M., Akitomo T., Kametani M., Kusaka S., Mitsuhata C., Nomura R. (2023). Dens invaginatus of fourteen teeth in a pediatric patient. Pediatr. Dent..

[54] Akitomo T., Ogawa M., Kaneki A., Nishimura T., Usuda M., Kametani M., Kusaka S., Asao Y., Iwamoto Y., Tachikake M. (2024). Dental Abnormalities in Pediatric Patients Receiving Chemotherapy. J. Clin. Med..

[55] Joffe S., Fernandez C.V., Pentz R.D., Ungar D.R., Mathew N.A., Turner C.W., Alessandri A.J., Woodman C.L., Singer D.A., Kodish E. (2006). Involving children with cancer in decision-making about research participation. J. Pediatr..

[56] Hein I.M., De Vries M.C., Troost P.W., Meynen G., Van Goudoever J.B., Lindauer R.J. (2015). Informed consent instead of assent is appropriate in children from the age of twelve: Policy implications of new findings on children’s competence to consent to clinical research. BMC Med. Ethics.

[57] Andreasen J.O., Borum M.K., Jacobsen H.L., Andreasen F.M. (1995). Replantation of 400 avulsed permanent incisors. 1. Diagnosis of healing complications. Endod. Dent. Traumatol..

[58] Sakai V.T., Moretti A.B., Oliveira T.M., Silva T.C., Abdo R.C., Santos C.F., Machado M.A. (2008). Replantation of an avulsed maxillary primary central incisor and management of dilaceration as a sequel on the permanent successor. Dent. Traumatol..

[59] Kocadereli I., Turgut M.D. (2005). Surgical and orthodontic treatment of an impacted permanent incisor: Case report. Dent. Traumatol..

[60] Cozza P., Marino A., Condo R. (2005). Orthodontic treatment of an impacted dilacerated maxillary incisor: A case report. J. Clin. Pediatr. Dent..

[61] Coste S.C., Silva E.F.E., Santos L.C.M., Barbato Ferreira D.A., Côrtes M.I.S., Colosimo E.A., Bastos J.V. (2020). Survival of Replanted Permanent Teeth after Traumatic Avulsion. J. Endod..

[62] Trope M. (1996). Protocol for treating the avulsed tooth. J. Calif. Dent. Assoc..

